# Draft Genome Sequence of Ligilactobacillus salivarius TUCO-L2, Isolated from Lama glama Milk

**DOI:** 10.1128/MRA.00784-20

**Published:** 2020-08-06

**Authors:** Leonardo Albarracin, Sandra Quilodrán-Vega, Kohtaro Fukuyama, Mikado Tomokiyo, M. Aminul Islam, Lorena Arce, Haruki Kitazawa, Julio Villena

**Affiliations:** a Laboratory of Immunobiotechnology, Reference Centre for Lactobacilli (CERELA-CONICET), Tucumán, Argentina; b Laboratory of Food Microbiology, Faculty of Veterinary Sciences, University of Concepción, Chillán, Chile; c Food and Feed Immunology Group, Laboratory of Animal Products Chemistry, Graduate School of Agricultural Science, Tohoku University, Sendai, Japan; d Livestock Immunology Unit, International Education and Research Center for Food and Agricultural Immunology (CFAI), Graduate School of Agricultural Science, Tohoku University, Sendai, Japan; Broad Institute

## Abstract

Ligilactobacillus salivarius TUCO-L2 was isolated from llama milk in Bio-Bio, Chile, and sequenced with the Illumina MiSeq platform. TUCO-L2 genome sequencing revealed a genome size of 1,600,747 bp with 1,691 protein-coding genes and a GC content of 33%. This draft genome sequence will contribute to a better understanding of the microbiome of llama milk.

## ANNOUNCEMENT

Maternal milk contains several species of lactobacilli (1, 2), including Ligilactobacillus salivarius (basonym Lactobacillus salivarius) (3), that beneficially modulate the establishment of gut microbiota and the development of immune systems in human (4–7), porcine, and bovine (8, 9) newborns. The milk microbiota from other domestic animals has been less explored. The Andean Mountains in South America harbor domestic animals with unique characteristics, including the domesticated camelid llama (Lama glama), which is valued in the local economy because of their meat, milk, and hair fiber (10, 11).

Here, we present the draft genome sequence of *L. salivarius* TUCO-L2, isolated from a milk sample from a llama in the Bio-Bio region of Chile. The milk sample (150 μl) was placed in de Man-Rogosa-Sharpe (MRS) broth (Oxoid, Cambridge, UK; pH 3; HCl, 5 N) at 37°C and 5% CO_2_ for 12 h, and then the cultures were transferred to MRS agar for colony isolation. A single colony of TUCO-L2 cultured on MRS agar plates was inoculated into MRS broth and incubated at 37°C for 12 h. Genomic DNA isolation was performed by using a lysozyme lysis buffer (75 mM NaCl, 20 mM EDTA, 20 mM Tris-HCl [pH 7.5], and 10 mg/ml lysozyme), the chloroform-isoamyl alcohol separation method, and the isopropanol precipitation method, as described previously (12). The genomic DNA of *L. salivarius* TUCO-L2 was sequenced with the Illumina MiSeq platform using the 2 × 300-bp paired-end read length sequencing protocol. The paired-end sequencing library was prepared using the TruSeq DNA high-throughput (HT) sample prep kit (Illumina) according to the manufacturer’s protocol. The paired-end reads were filtered with PrinSeq lite v0.20.4 to remove low-quality reads (-min_qual_mean, 20; –min_len, 75) (13). After filtering, the selected reads were assembled using SPAdes v3.11.1 (14) with default parameters. The sequencing protocol generated a mean genome coverage of 52×. The TUCO-L2 draft genome sequence contains 409 contigs with an average GC content of 33% and a total estimated size of 1,600,747 bp.

Sequencing results were analyzed using various software programs at their default settings, unless otherwise specified. Gene prediction and annotation were performed using NCBI Prokaryotic Genome Annotation Pipeline (PGAP) v4.8 (15) and Rapid Annotations using Subsystems Technology (RAST) (16). This genome contained 1,691 DNA-coding sequences, 17 tRNA-coding sequences, and 3 rRNA-coding sequences.

System category distribution by RAST showed 210 subsystems. The TUCO-L2 genome contains genes involved in the transport and metabolism of lactose, glucose, and galactose that would confer to the bacteria the capacity to survive in the milk. A gene for a cholylglycine hydrolase was also detected, which could be involved in its ability to survive in the gastrointestinal tract (17). Genes of the SecA2-SecY2 system and two genes of MucBP domain-containing proteins were found in the genome of TUCO-L2, which have been proposed as important factors for the adaptation to the intestinal tract in *L. salivarius* strains isolated from pigs and chickens (17). BAGEL4 (18) and CRISPRCasFinder (19) were used to analyze the presence of bacteriocins and clustered regularly interspaced short palindromic repeats (CRISPR), respectively. The analysis revealed the presence of the enterolysin A gene and 1 CRISPR array in the draft genome sequence.

The draft genome sequence of *L. salivarius* TUCO-L2 will be useful for further studies of specific genetic features and could contribute to a better understanding of the microbiome of *L. glama* milk.

### Data availability.

This whole-genome shotgun project has been deposited at DDBJ/ENA/GenBank under the accession number SOPE00000000. The version described in this paper is the first version, SOPE01000000. The SRA/DRA/ERA accession number is ERP114188. The BioProject and BioSample numbers are PRJNA527825 and SAMN11160677, respectively.
